# Editorial: Neuroendocrine tumors: the road to precision medicine

**DOI:** 10.3389/fendo.2023.1253319

**Published:** 2023-07-26

**Authors:** Anna La Salvia, Dario Giuffrida, Roberta Modica

**Affiliations:** ^1^National Center for Drug Research and Evaluation, National Institute of Health (ISS), Rome, Italy; ^2^Department of Oncology, Istituto Oncologico del Mediterraneo, Viagrande, Catania, Italy; ^3^Unit of Endocrinology, Department of Clinical Medicine and Surgery, University of Naples Federico II, Naples, Italy

**Keywords:** neuroendocrine neoplasm (NEN), pancreas, medullary thyroid carcinoma, Merkel cell carcinoma, insulinoma, personalized medicine

Precision medicine is growing as an innovative approach in the neuroendocrine field, aiming to obtain a detailed understanding of the disease, including epigenetic and omics characterization, to ultimately improve patient care (1, 2). Neuroendocrine neoplasms (NEN) are heterogeneous tumors with increasing incidence, showing some common pathological features and variable clinical presentation and outcomes (3). Since the availability of somatostatin analogs, the therapeutic options have considerably expanded, taking into account immunotherapies and metabolic profiling (4–7). Risk and prognostic factors are being analyzed in detail, along with a comprehensive evaluation of quality of life (8–11).

The current Research Topic, “*Neuroendocrine tumors: the road to precision medicine*”, in the Frontiers in Endocrinology Journal, is dedicated to collecting high-quality scientific contributions that mainly focus on recent advances in the context of the diagnosis, treatment, and prognosis of NETs.

With these premises, two studies in the current Research Topic present an analysis of epidemiological data collected from the Surveillance, Epidemiology, and End Results (SEER) database in two challenging subtypes of NET; Merkel cell carcinoma (MCC), a highly aggressive neuroendocrine carcinoma, which correlates with poor prognosis; and functional pancreatic NET (F-PNETs), a heterogeneous group of pNET associated with the clinical diagnosis of hormonal hypersecretion and syndrome. In both cases, the main aim is to assess and further predict patients’ survival outcomes. Specifically, the work by Xu W et al. elaborates an easy-to-use web-based calculator to predict the overall survival of MCC patients. This tool is based on a nomogram including key prognostic variables. The study by Luo S et al. shows that F-PNETs incidence decreased over the study period (2000-2017). In addition, the statistical Cox proportional hazards model identifies tumor size, tumor stage, tumor type, and surgical resection as the main prognostic factors for F-PNETs.

One study in the current Research Topic, by Fanciulli, is focused on the assessment of a new potentially relevant class of drugs for medullary thyroid cancer (MTC), proteasome inhibitors (PrIn). Fanciulli G et al. summarize the available *in vitro* and *in vivo* data on the role of PrIn as monotherapy as well as in combination with other treatments for MTC. Their review highlights that despite the encouraging preclinical activity of PrIn in *in vitro* studies in human and murine MTC cell lines, evidence in the clinical setting is currently scarce. The authors advocate the launch of new clinical trials to establish the potential usefulness of these agents for MTC.

Finally, two case reports are published within this Research Topic. Sira L et al. describe the case of a young woman with non-functioning advanced pNET associated with the mutation of JAK2V617F, which is an independent factor for thromboembolic events. The patient presented at first with massive portal and splenic vein thrombosis, requiring anticoagulation treatment. Three years later, she was diagnosed with a grade 2 (Ki-67 index of 6%) pNET, associated with unresectable liver metastases. In this case, the surgical removal of the pancreatic tumor was performed and systemic treatment with somatostatin analogs (SSA) was started. The treatment was stopped for the duration of the patient’s two pregnancies and then started again. Notably, after parturition, a complete remission of the liver lesions was achieved. Whole Exome Sequencing (WES) was performed but failed to demonstrate a mutation in MEN1, VHL, NF1, TSC1, TSC2, MUTYH, BRCA2, and CHEK2 genes. The second case report, by Tarris G et al., describes a giant (88 x 73 mm) F-PNET in a young woman. The diagnostic work-up was started due to the onset of hypoglycemia unresponsive to treatment with diazoxide. A CT scan detected the mass located in the pancreatic tail, which was surgically removed. At the histopathological evaluation, a grade 1 (Ki-67 < 2%) pNET with cytoplasmic expression of insulin in tumor cells was found, confirming the diagnosis of insulinoma. After a 16-month follow-up period after pancreatic surgery, the clinical symptoms were recovered and the patient was found to be free from disease recurrence.

In conclusion, this Research Topic highlights recent advances in precision medicine in NET, including rare primaries such as MCC and MTC. The various contributions in this Research Topic demonstrate how the development of patient-tailored treatment approaches is evolving in the neuroendocrine field. This will likely result in better patient care and survival thanks to personalized treatments. We believe this Research Topic has the potential to support specialists involved in treating NEN.

## Author contributions

ALS, DG, and RM contributed to the development of this article by summarizing the results of all scientific manuscripts included in the Research Topic “*Neuroendocrine tumors: the road to precision medicine*”. All authors contributed to the article and approved the submitted version.
